# Women’s Access to Abortion Care Under Oregon's Reproductive Health Equity Act

**DOI:** 10.1001/jamahealthforum.2021.0402

**Published:** 2021-05-21

**Authors:** Maria I. Rodriguez, Megan Skye, Mitra Shokat, Rachel Linz, Nisreen Pedhiwala, Blair G. Darney

**Affiliations:** 1 Department of Obstetrics and Gynecology, Oregon Health & Science University, Portland; 2 School of Public Health, Oregon Health & Science University–Portland State University, Portland; 3 Reproductive Health Program, Oregon Health Authority, Salem

## Abstract

This cohort study describes the first 24 months of abortion services covered under Oregon’s Reproductive Health Equity Act and distances traveled by women to receive care.

## Introduction

Unintended pregnancy and abortion are becoming increasingly concentrated among women in disadvantaged communities who experience substantial barriers to obtaining health care.^1^ A common barrier to accessing needed abortion care is cost, as more than half of all abortions are paid for out of pocket.^2^ While no federal funds can be used for abortion, 16 states, including Oregon, use state funds to cover all or most abortions in Medicaid.^3^ However, federal law restricts access to Medicaid for undocumented and recent immigrants.^4^

Oregon’s Reproductive Health Equity Act (RHEA) took effect January 1, 2018, and ensured coverage for family planning (abortion and contraception) using state funds for all low-income state residents regardless of citizenship status. We describe the first 24 months of abortion services covered under RHEA and distances traveled by women to receive care.

## Methods

We conducted a cross-sectional study of abortion services that were reimbursed under RHEA in 2018 and 2019. We used data from the program’s “Clinic Visit Record,” which includes demographic and medical information, as well as billing claims. This program is separate from the state’s Medicaid program. We included abortions that were reimbursed under RHEA at 11 clinics statewide that provide abortion services, with the exception of 1 hospital-based clinic. No hospital was contracted to provide scheduled abortion care during the study period. Emergency hospital-based abortions for life-threatening conditions (eg, hemorrhage) would be reimbursed by Medicaid and are not included in the study sample. This study followed the Strengthening the Reporting of Observational Studies in Epidemiology (STROBE) reporting guideline. The institutional review boards at Oregon Health & Science University and the Oregon Health Authority approved the study and granted a waiver of informed consent based on the lack of HIPPA identifiers.

We describe sociodemographic, clinical, and abortion characteristics of women who received abortions under RHEA by metropolitan residence (vs nonmetropolitan) based on zip code. Our primary outcome was the distance traveled to receive an abortion. We used the patient-reported zip code of residence to calculate the distance the patient traveled to receive an abortion using the straight-line distance between centroids of the patient’s zip code and the latitude and longitude of the clinic where the patient received an abortion. Statistical analyses were conducted using Stata, version 16 (StataCorp), and statistical significance was set at *P *< .05.

## Results

Oregon’s RHEA provided access to 625 clinic-based abortions. Nearly half (302 [48.3%]) of the abortions were for women aged 25 to 34 years (range, 15-46 years). Most abortions (498 [79.7%]) were for women who resided in metropolitan zip codes. Consistent with national trends, most abortions (583 [93.9%]) occurred during the first trimester, and slightly more than half (358 [57.3%]) were surgical abortions.^5^ More than 509 abortions (80%) were performed for women who had previously given birth. There was no difference in rates of second trimester abortion by residence (5.6% metropolitan vs 6.3% nonmetropolitan; *P* = .94).

In the overall cohort, the median distance traveled for an abortion was 8.73 miles (interquartile range, 4.78-17.20 miles; range, 0.42-124.44 miles). A third of women (189 [30.2%]) traveled less than 5 miles to receive abortion care, and 32 (5.1%) traveled 50 or more miles.

## Discussion

In the first 2 years following RHEA implementation, immigrant women across the state used the expanded coverage to access abortion, indicating that the policy was fully implemented in metropolitan and nonmetropolitan areas. The distances traveled for abortion in Oregon were lower than national averages (5.1% of RHEA clients vs 18% nationally traveling more than 50 miles).^5^ Our study is limited by the lack of information on abortions received by low-income immigrants before implementation of the RHEA policy and not including information on contraception. State policies, such as RHEA, can ensure that low-income individuals are not excluded from family planning services based on citizenship status.
